# Immunotherapy in Advanced Gastric Cancer: An Overview of the Emerging Strategies

**DOI:** 10.1155/2018/2732408

**Published:** 2018-06-20

**Authors:** Helena Magalhães, Mário Fontes-Sousa, Manuela Machado

**Affiliations:** Medical Oncology Department, Portuguese Institute of Oncology of Porto (IPO Porto), Porto, Portugal

## Abstract

Gastric cancer (GC) remains a public health problem, being the fifth most common cancer worldwide. In the western countries, the majority of patients present with advanced disease. Additionally, 65 to 75% of patients treated with curative intent will relapse and develop systemic disease. In metastatic disease, systemic treatment still represents the state of the art, with less than a year of median overall survival. The new molecular classification of GC was published in 2014, identifying four distinct major subtypes of gastric cancer, and has encouraged the investigation of new and more personalized treatment strategies. This paper will review the current evidence of immunotherapy in advanced gastric cancer.

## 1. Introduction

GC is the 5th most common cancer diagnosed worldwide, and it represents one of the major causes of malignant disease morbidity and mortality, with almost 107,000 deaths in Europe in 2012 [1, 2].

The majority of the patients are diagnosed with locally advanced disease not suitable for surgery or metastatic disease. For these patients, chemotherapy is the standard of care in patients with clinical conditions, with median overall survival (OS) of less than 12 months. When compared to best supportive care (BSC), systemic treatment showed a clear advantage in OS [3, 4].

Currently, a combination of a platinum and fluoropyrimidine doublet is the mainstay of chemotherapy. The addition of a taxane or an anthracycline to this combination in human epidermal growth factor receptor 2 (HER-2) negative population increases response rate and survival outcomes but also generally implies higher toxicity, so the risks versus benefits should be well balanced. In the phase III ToGA trial, the addition of trastuzumab to cisplatin and fluoropyrimidine backbone improved median overall survival (OS), progression free survival (PFS), and response rate (RR) in Her-2 positive advanced or metastatic gastric cancer and established this regimen as standard of care in those patients [5, 6].

Second line chemotherapy, is an option for patients with good performance status. Docetaxel, irinotecan, and paclitaxel have all demonstrated improved survival compared to BSC in this setting. Additionally, ramucirumab, a vascular endothelial growth factor receptor (VEGFR-2) antibody, was the first biological treatment given as a single drug or in combination with paclitaxel in patients with advanced gastric or gastroesophageal junction (GEJ) adenocarcinoma progressing after first-line chemotherapy that demonstrated survival benefits in two randomized trials [7–11].

Despite these treatment options, the prognosis of advanced and metastatic GC is still poor and novel treatment strategies and patient selection tools are needed.

In the “era of the revolution” in cancer management with immunotherapy, it appears that a new hope is also arising for patients with advanced GC, as it has in other malignancies where this class of drugs demonstrated benefit.

Evidence and rationale for the use of immunotherapy in gastric cancer GC is a heterogeneous disease which can be divided into 4 major subtypes based on molecular signature according to Cancer Genome Atlas Research Network (TCGA): Epstein Barr virus (EBV) positive, microsatellite unstable (MSI), and genomically stable (GS) and chromosomal instability (CIN) tumours [12].

Two subtypes, EBV positive and MSI GC, are considered to be most potentially responsive to immunotherapy drugs.

The EBV positive GC that represents 9% of all GC is more prevalent in younger patients, in males (a twofold ratio in male/female), with no difference between intestinal and diffuse histology. EBV positive GC is associated with programmed death-ligand 1 (PD-L1) gene amplification, which suggests higher immunogenicity, and might therefore be more likely to respond to immune checkpoint inhibition. It is known that PD-L1 is highly predictive in lung cancer, but yet controversial in gastric cancer.

MSI tumours seem to occur in 15–30% of GC and are related more commonly with female gender, older patients, and intestinal histology and tumours arising from the distal stomach. This category of gastric cancer is characterized by increased lymphocytic infiltrate, which may reflects activation of T-cells against tumour antigens and genomic changes in tumour cells that are linked to PD-L1 expression, indicating a potential role for immunotherapy [13].

Both MSI and EBV positive GCs have a high somatic mutational burden which also is a feature that has been associated with response to immunotherapy.

## 2. Checkpoint Inhibition

Given the success of checkpoint inhibitors in melanoma, non-small-cell lung cancer, renal cell cancer, urothelial carcinoma, and head and neck cancer it seemed logical to investigate the role of these agents in gastric cancer.

Cytotoxic T lymphocyte protein 4 (CTLA-4) and programmed cell death protein-1 (PD-1) are immune checkpoints that inhibit the T-cell response, which provide the escape mechanism of the tumour cells to T-cell antitumour activity [14].

The B7-H1, also known as PD-L1, in positive tumours interacts with its receptor PD-1 and this consequently leads to inhibition of the T-cells migration, proliferation, resulting in an antiapoptotic signal, preventing overactivation of the immune system, escaping from destruction [15].

In GC, some studies evaluate the expression and clinical significance of PD-1/PD-L1 pathway. Wu et al. found that PD-L1 was expressed in 42,2% of GC tissues and was not found in normal tissue. The immunodetection of PD- L1 was significantly associated with tumour size, invasion, lymph node metastasis, and survival time of patients [16].

In another study, Hou et al. found the expression of PD-L1 in 63% of the 111 GC patients analyzed and that its overexpression was linked to lymph node metastasis, an advanced clinicopathological stage, and lower overall survival rate [17].

**Figure 1:**
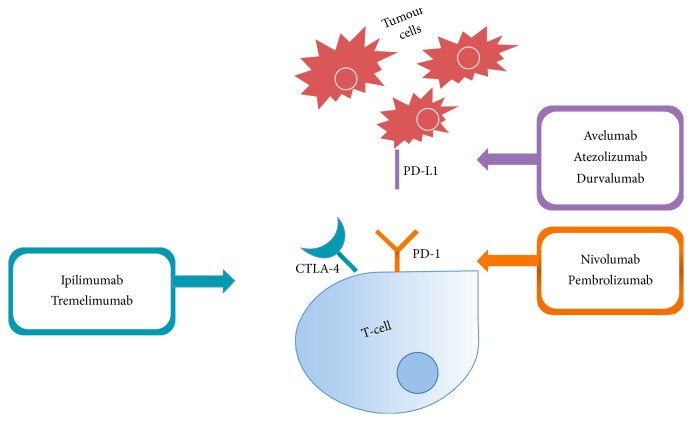
Immune checkpoint blockade with different monoclonal antibodies.

Therefore, immunologic checkpoint blockade with antibodies that target CTLA-4 and PD-1/PD-L1 seemed promising strategies that could improve the outcomes in GC and deserved more specific studies (Figure 1).

We tried to summarize the relevant clinical data about specific immune checkpoints agents and the possible future applications in treatment of advanced gastric cancer.

## 3. Anti-CTLA4

Ipilimumab and tremelimumab are two anti-CTLA4 antibodies that were evaluated in GC.

A phase II trial evaluated the efficacy of ipilimumab immediately following 1st line chemotherapy in unresectable or metastatic adenocarcinoma of the gastric and GEJ compared with BSC. From 143 patients screened, 57 were randomized to each arm, and in an interim analysis, no differences were seen in PFS between groups, and the study ended early. At study closeout (8 months after interim analysis), the median OS was 12.7 months in BSC versus 12.1 months for the arm with ipilimumab [18].

Tremelimumab was investigated in a phase II trial as 2nd line treatment for patients with metastatic gastric and oesophageal adenocarcinomas. The response rate was only 5%, but there was a clinical benefit with evidence of stable disease in 4 of the 18 patients, and one patient showed a durable response, receiving 32.7 months of treatment after trial enrollment [19].

## 4. Anti-PD-1

Nivolumab is a PD-1 blocking antibody approved for the treatment of advanced melanoma, advanced non-small-cell lung cancer (NSCLC), advanced renal cell carcinoma, advanced squamous cell carcinoma of the head and neck (SCCHN), and urothelial carcinoma.

Two randomized trials showed efficacy and safety for nivolumab alone in both Asian and western populations in gastric cancer.

The phase I/II CHECKMATE 032 trial, a multicohort study, included patients with metastatic gastric or GEJ cancer, treated with nivolumab in monotherapy (3 mg/kg IV every 2 weeks) or in combination with ipilimumab, irrespective of PD-L1 status [20].

In the single-arm (the cohort with 59 patients), the objective response rate, defined as the proportion of patients who achieved a complete response or a partial response (ORR), with nivolumab was 14% (including 1 complete response and 7 partial responses). Moreover, the stable disease rate was 19%, for a total disease control rate of 32%. The median time to response was 1.6 months and the median duration of response was 7.1 months. The median OS was 5.0 months with nivolumab (95% CI, 3.4–12.4). The 12-month OS rate was 36%. The median PFS was 1.36 months (95% CI, 1.3–1.5) and the 6-month PFS rate was 18%. In the subgroup with PD-L1 expression on ≥1% of cells (*n* = 15), the ORR was 27% with nivolumab. In those with PD-L1 expression on <1% (*n* = 25), the ORR was 12%.

The combination of nivolumab with ipilimumab was also evaluated in this trial, with two separate dose levels: nivolumab 1 mg/kg and ipilimumab 3 mg/kg (*n* = 49) or nivolumab 3 mg/kg plus ipilimumab 1 mg/kg (*n* = 52). The ORR was 26% for the first arm and 10% for the second. Six-month PFS was 24% and 9%, respectively. The 12-month OS was 34% in first cohort and not available in the second. Grade 3 or greater adverse effects (AEs) were seen in 27 and 45% of the patients, respectively, which was higher than in the nivolumab alone arm (17%).

The ONO-4538-12 ATTRACTION-2 trial evaluated the efficacy and safety of nivolumab in Asian patients with unresectable advanced or recurrent gastric cancer (including GEJ) who progressed after two or more chemotherapy lines of treatment [21].

Median OS was 5.26 months (95% CI = 4.60–6.37) for patients treated with nivolumab, compared to 4.14 months (95% CI = 3.42–4.86) for those treated with placebo.

In addition, the 12-month OS in the nivolumab group was 26.2% (95% CI = 20.7–32.0) versus 10.9% (95% CI = 6.2–17.0) in the placebo group. Patients treated with nivolumab had an ORR of 11.2% (95% CI 7.7–15.6) compared to 0% (95% CI 0.0–2.8) with placebo. Patients with confirmed response to nivolumab had a median duration of response of 9.53 months (95% CI 6.14–9.82). Grade 3 or greater AEs occurred in 10% of nivolumab arm and 4% of placebo arm.

There were divergent results according to tumour negative PD-L1 expression versus ≥1%. In tumour with negative PD-L1 expression, median OS was 6.05 months in nivolumab arm (versus 4.19 months in the placebo arm; hazard ratio 0.72); in patients with PD-L1 expression ≥1%, median OS was 5.22 months in the arm of nivolumab (versus 3.83 months in the placebo arm; hazard ratio 0.51).

Currently, an important milestone marked the oncology community: pembrolizumab, a humanized IgG4 monoclonal anti-PD1 antibody, had accelerated approval by FDA (Food and Drug Administration) for the treatment of adult patients with unresectable or metastatic solid tumours that have been identified as having a biomarker referred to as microsatellite instability-high (MSI-H) or mismatch repair deficient (dMMR). This indication includes patients that have progressed following prior treatment and who have no satisfactory alternative treatment options.

Results on safety and efficacy from pembrolizumab specifically in gastric cancer were first presented at ESMO Congress 2014 by Muro et al. (KEYNOTE-012) and published in 2016 [22, 23]. Of the 39 patients included in gastric cancer cohort, the ORR was 22% (95% CI 10–39) by central review, all partial responses. Median time to response was 8 weeks (range 7–16), with median response duration of 24 weeks. The 6-month PFS rate was 26% (95% CI 13–41) and OS rate was 66% (95% CI 49–78) and 42% (95% CI 25–59) at 6 and 12 months, respectively. The toxicity was manageable, with only 5 patients experiencing grade 3 or greater adverse effects.

KEYNOTE-059 is a phase II trial multicohort study in advanced gastric or GEJ adenocarcinoma. In cohort 1, patients who have received at least two prior therapies received pembrolizumab as monotherapy. In cohort 2 patients who have not received any previous therapy for their disease received pembrolizumab in combination with cisplatin and 5-FU (in Japan capecitabine could be used instead of 5 FU). In Cohort 3, participants who did not received any previous therapy and who had PD-L1 positive tumours received monotherapy with pembrolizumab.

The results of cohort 1 were presented at ASCO 2017 and the updated data was also presented at ESMO 2017 [24, 25]. From 259 patients in cohort 1, 76.4% were male, and median age was 62.0 years, with patients from United States (47.9%), East Asia (13.1%), and the rest of the world (39.0%); 51.7% and 29% of the patients received pembrolizumab as 3rd line (3L) and 4th line therapy, respectively.

PD-L1 positive patients had expression in ≥1% tumour or stromal cells using immunohistochemistry (IHC). In this cohort 57.1% had PD-L1 positive tumours.

The ORR with pembrolizumab in all patients was 11.6% (95% CI 8.0–16.1). In PD-L1-positive ORR was 15.5% (95% CI 10.1–22.4) and in PD-L1 negative tumours ORR was 6.4% (95% CI 2.6–12.8). The median duration of response (DOR) in all patients was 8.4 months. The median DOR in the PD-L1–positive group was 16.3 versus 6.9 months in those with PD-L1–negative disease.

In the 7 patients with MSI-H tumours, ORR was 57.1%; in comparison with 167 patients with non-MSI-H tumours, ORR was 9.0%.

The median PFS was 2.0 months and the median OS was 5.6 months. Treatment was well tolerated, but 2 treatment-related grade 5 AEs were reported (acute kidney injury and pleural effusion).

In 3rd line the ORR was 16.4% (95% CI 10.6–23.8), with 3% of CR and 13.4% of PR; in 4th line the ORR was 6.4% (95% CI 2.8–12.2).

The cohort 2 was presented in ASCO 2017 and the updated data was also presented at ESMO 2017 [25, 26]. From 25 enrolled patients, 64% were men, median age was 64 years, 68% were Asian, and 64% had PD-L1 positive tumours. In PD-L1-expressing patients, ORR was 68.8% versus 37.5% in PD-L1-negative patients. Median duration of response was 4.6 months in overall population, 4.6 months in PD-L1-positive patients, and 5.4 months in PD-L1-negative patients. Investigators observed grade 3/4 AEs in 76% of patients.

The cohort 3 was presented at ESMO congress in September 2017 [25]. In the 31 patients included, with a median follow-up of 17.5 months, the ORR was 26% and the DCR 36%. The median PFS was 3.3 months and the median OS 20.7 months.

Several randomized clinical trials are currently ongoing to evaluate pembrolizumab and nivolumab in earlier lines of therapy in monotherapy and in combination with chemotherapy regimens or biologic agents for patients with advanced gastric/gastroesophageal cancer (Table 1).

## 5. Anti-PD-L1

Avelumab is a fully human anti-PD-L1 IgG1 antibody, and its efficacy and safety were first investigated in a phase 1b trial, in patients with advanced gastric or GEJ in first line as maintenance and in second line (2L) of treatment. Patients received avelumab at 10 mg/kg IV every 2 weeks until progression, unacceptable toxicity, or withdrawal [27].

The ORR, until now unconfirmed, in maintenance and 2L was 7.3% (with 1 complete response, 3 partial responses) and 15%, respectively. The disease control rate (DCR) was 54.5% and 50%, and median PFS was 14,1 and 11,6 weeks in two arms (maintenance and 2L respectively). A trend towards longer PFS was observed in patients with PD-L1-positive tumours. Grade ≥ 3 AEs were documented in 9.9% patients, which included fatigue, asthenia, increased gamma-glutamyl transferase (GGT), thrombocytopenia, and anaemia. There was 1 treatment-related death (hepatic failure/autoimmune hepatitis).

With these encouraging results, two randomized trials with avelumab were envisaged: JAVELIN Gastric 300 (NCT02625623) that will compare avelumab plus BSC in third line treatment versus physician's choice of chemotherapy plus BSC and JAVELIN Gastric 100 (NCT02625610), a phase 3 trial, whose purpose is to demonstrate the superiority of treatment with avelumab as maintenance versus continuation of first-line chemotherapy with oxaliplatin-fluoropyrimidine doublet.

Durvalumab is a humanized IgG-1*κ* monoclonal antibody that blocks PD-L1.

Segal et al. reported durvalumab clinical activity in an expansion study in multiple cancer types, including NSCLC, melanoma (cutaneous and ocular), gastroesophageal, hepatocellular carcinoma, pancreatic, SCCHN, and triple negative breast cancer. Durvalumab was administered as 10 mg/kg IV every 2 weeks for 12 months. This agent showed clinical activity in gastric cancer with an ORR of 25% (4 partial responses). Treatment-related AEs occurred in one-third of the patients, with ≥Grade 3 AEs in 7% and none led to discontinuation of study drug [28].

Durvalumab, as maintenance, as in combination with a variety of immunomodulators and targeted agents is ongoing in gastric cancer field (Table 2).

Atezolizumab is another human monoclonal antibody that contains an engineered Fc-domain that targets PD-L1, blocking PD-L1 from binding to PD-1 and B7.1, and demonstrated clinical activity in locally advanced and metastatic cancers. In a phase I trial, atezolizumab was administered as a single agent to patients with locally advanced or metastatic solid tumours or hematologic malignancies, and 175 patients were evaluated by RECIST v1.1 and confirmed that complete and partial responses were observed in 18% of patients with all tumour types, 21% in NSCLC, 26% in melanoma, 13% in renal cell carcinoma, and 13% of patients with other tumours including colorectal cancer, gastric cancer (only one patient), and head and neck squamous cell carcinoma. A statistical association between tumours expressing high levels of PD-L1 was observed, especially PD-L1 expressed by tumour-infiltrating immune cells and response to atezolizumab treatment [29, 30].

## 6. Discussion

After a long time of stagnation in GC treatment, with only two molecular target agents providing modest results in OS and PFS (trastuzumab and ramucirumab), maybe a new paradigm shift in oncology is arising: instead of targeting cancer cells, we can target immune cells, thus stimulating the host immune system against its own cancer cells [31].

Gastric cancer is a heterogeneous condition stratified in 4 molecular subtypes, based on genomic changes [12]. The molecular classification improved our knowledge about the biologic behavior of this disease and offered potential actionable oncogenic drivers. With this deep understanding, we will maximize treatment efficacy.

Certainly, MSI and EBV subtype are of particular interest, deriving from their high immunogenicity and potential greater response with immunotherapy agents.

As detailed before, immune checkpoint blockade with antibodies targeting CTLA-4, PD-1, and PD-L1 has revealed clinical activity in gastric cancer. While anti-CTLA4 showed only slight activity in gastric cancer, and PD-1 and PD-L1 inhibitors showed promising results and will probably take place in gastric cancer management in the near future.

We would like to highlight the phase III KEYNOTE-059 trial, as it showed antitumour activity and durable responses in patients with advanced gastric/GEJ cancer progression after more than 2 lines of therapy. Until now there was no evidence for 3rd and 4th lines in gastric cancer, and based on the cohort 1 results, pembrolizumab was approved by the FDA recently [24].

In the cohort 2, patients received pembrolizumab and chemotherapy with cisplatin and 5- fluorouracil, with favourable clinical activity and manageable toxicity, though more data is needed to draw conclusions [25, 26].

Also, a question to consider is if the results of nivolumab in Asian patients will be reproduced in western patients? [20, 21]

Results from phase I/II CheckMate 032 trial, which included heavily pretreated European and North American population, revealed long-term overall survival and responses with nivolumab. These findings suggest a possible benefit with nivolumab in Asian and western patients, although we need more studies to make a definitive conclusion.

This and much more questions remain to be answered: which gastric cancer subpopulation does benefit more from immune checkpoints inhibitors? In which stage of the disease should we use immunotherapy, in earlier lines or after progression of more than 2 lines of therapy?

We look forward at the ongoing phase III trials and wait with hope for their results. Besides, more studies are needed to validate predictive and prognostic biomarkers to immunotherapy agents in gastric cancer.

Additionally, integration of immune checkpoints combined with targeted agents, chemotherapy, or radiotherapy appears to be exciting multimodal approaches and randomized trials are also ongoing.

In conclusion, some progress has been reached in the treatment of advanced gastric cancer in the last years.* *With the recent biologic and molecular knowledge, we have recognized that gastric cancer is a group of distinct molecular entities rather than a single disease. This molecular characterization will allow achieving a better selection of patients that can benefit from a treatment strategy.

The field is unquestionably moving towards a more precise medicine, and the progressing accomplishments will transform the clinical practice in the management of advanced gastric cancer in the near future.

## Figures and Tables

**Table 1:** Ongoing trials with anti-PD1 in advanced gastric cancer.

Study ID	Study phase	Treatment	Population	Status
NCT02901301	Ib/II	Pembrolizumab + trastuzumab + cisplatin + capecitabine	HER2 positive advanced gastric cancer	Recruiting

CP-MGAH22-05 (NCT02689284)	Ib/II	Margetuximab in combination with pembrolizumab	Relapsed/refractory advanced HER2+ GEJ or gastric cancer	Recruiting
NCT02318901	Ib/II	Pembrolizumab and monoclonal antibody therapy	Patients with advanced cancer (one cohort for patients with unresectable HER2 overexpressing gastric or GEJ cancers)	Active, not recruiting
NCT03095781	Ib	Pembrolizumab and XL888	Patients with stage IV or locally advanced unresectable gastrointestinal adenocarcinomas who have failed at least one prior therapy	Recruiting

NCT02178722	I/II	Pembrolizumab in combination with epacadostat	Patients with selected cancers (including gastric cancer)	Recruiting

NCT03342937	II	Pembrolizumab + oxaliplatin and capecitabine	First-line treatment of patients with gastroesophageal cancer	Not yet recruiting

NCT02954536	II	Pembrolizumab in combination with trastuzumab, capecitabine/cisplatin	First-line stage IV HER2-positive metastatic esophagogastric (EG) cancer	Recruiting

NCT03196232	II	Epacadostat and pembrolizumab	Metastatic or unresectable GEJ or gastric cancer that progressed at least first line of prior therapy	Recruiting

KEYNOTE KN-463 (NCT03122548)	II	CRS-207 and pembrolizumab	Recurrent or metastatic gastric, GEJ, or esophageal cancer who have received 2 prior systemic chemotherapy treatment	Recruiting

KEYNOTE-063 (NCT03019588)	III	Pembrolizumab versus paclitaxel	Asian subjects with advanced gastric or GEJ adenocarcinoma who progressed after first-line therapy with platinum and fluoropyrimidine	Recruiting

KEYNOTE-062 (NCT02494583)	III	Pembrolizumab as monotherapy and in combination with cisplatin + 5-fluorouracil versus placebo + cisplatin + 5-fluorouracil	As first-line treatment in subjects with advanced gastric or GEJ adenocarcinoma	Active, not recruiting

KEYNOTE-061 (NCT02370498)	III	Pembrolizumab versus paclitaxel	Advanced gastric or GEJ adenocarcinoma who progressed after first-line therapy with platinum and fluoropyrimidine	Active, not recruiting

ONO4538 (NCT02267343)	III	Nivolumab versus placebo	Unresectable advanced or recurrent gastric cancer (including esophagogastric junction cancer) refractory to or intolerant of standard therapy	Active, not recruiting
CA209-929 (NCT03342417)	II	Combination of nivolumab and ipilimumab in breast, ovarian, and gastric cancer patients	In gastric cancer arm: advanced gastric cancer patients who are recurrent/refractory to a prior therapy not involving herceptin	Recruiting
ONO-4538-37 (NCT02746796)	II/III	Nivolumab and chemotherapy versus placebo and chemotherapy	Unresectable advanced or recurrent gastric cancer (including esophagogastric junction cancer) not previously treated with the first-line therapy	Recruiting
CheckMate 649 (NCT02872116)	III	Nivolumab plus ipilimumab or nivolumab in combination with oxaliplatin plus fluoropyrimidine versus oxaliplatin plus fluoropyrimidine	Patients with previously untreated advanced or metastatic gastric or gastroesophageal junction cancer	Recruiting
FRACTION-GC (NCT02935634)	II	Nivolumab plus ipilimumab versus nivolumab plus relatlimab versus nivolumab and BMS-986205	Patients with advanced gastric cancer	Recruiting

NCCH-1611 NCT02999295	I/II	Ramucirumab plus nivolumab	Second-line therapy in Participants with gastric or GEJ cancer	Recruiting
AIO-STO-0217 (NCT03409848)	II	Ipilimumab or FOLFOX in combination with nivolumab and trastuzumab	Previously untreated HER2 positive locally advanced or metastatic esophagogastric adenocarcinoma	Not yet recruiting
INCAGN 1876-201 (NCT03126110)	I/II	INCAGN01876 combined with nivolumab versus INCAGN01876 combined with ipilimumab versus INCAGN01876 combined with nivolumab and ipilimumab	Subjects with advanced or metastatic malignancies	Recruiting

**Table 2:** Ongoing trials with anti-PD-L1 in advanced gastric cancer.

Study ID	Study phase	Treatment	Population	Status
YO39609 (NCT03281369)	I/II	Multiple immunotherapy-based treatment combinations, including atezolizumab as immunotherapeutic agent	Patients with locally advanced unresectable or metastatic gastric or gastroesophageal junction cancer	Recruiting

JAVELIN Gastric 300 (NCT02625623)	III	Avelumab + best supportive care (BSC) versus physician's choice chemotherapy + BSC or BSC alone	Unresectable, recurrent, locally advanced, or metastatic gastric or gastroesophageal junction adenocarcinoma gastric cancer third line	Active, not recruiting

JAVELIN Gastric 100 (NCT02625610)	III	Avelumab (MSB0010718C) versus continuation of first-line chemotherapy	Unresectable, locally advanced, or metastatic adenocarcinoma of the stomach or of the gastroesophageal junction	Active, not recruiting

JAVELIN MEDLEY (NCT02554812)	Ib/II	Avelumab (MSB0010718C) in combination with other cancer immunotherapies	Patients with locally advanced or metastatic solid tumors	Recruiting

MEDIOLA (NCT02734004)	I/II	MEDI4736 in combination with olaparib	Patients with advanced solid tumors, selected based on a rationale for response to olaparib	Active, not recruiting

I4T-MC-JVDJ (NCT02572687)	I	Ramucirumab plus MEDI4736	Participants with locally advanced and unresectable or metastatic gastrointestinal or thoracic malignancies including gastric or gastroesophageal junction (GEJ) adenocarcinoma, non-small-cell lung cancer (NSCLC) or hepatocellular carcinoma (HCC)	Active, not recruiting

PLATFORM (NCT02678182)	II	Maintenance therapies following completion of standard first-line chemotherapy: placebo versus capecitabine versus durvalumab versus trastuzumab versus rucaparib	Patients with locally advanced or metastatic HER-2 positive or HER-2 negative oesophagogastric adenocarcinomas	Recruiting

D419SC00001 (NCT02658214)	Ib	Durvalumab and tremelimumab in combination with first-line chemotherapy	Patients with advanced solid tumors	Recruiting

MEDI4736 also known as durvalumab.

## Additional Points

*Core Tip.* GC is a highly heterogeneous disease and the recent molecular characterization will help us to better select patients who might benefit from immune checkpoint inhibitors and other agents. There are encouraging results with agents that target programmed death 1 (PD-1) and its ligands in gastric cancer; however more trials are needed to identify predictive and prognostic biomarkers to select patients most appropriately for this treatment. In this review, we explore the current evidence supporting the use of immunotherapy in advanced GC.
